# Diagnosis Accuracy of Lung Ultrasound for ARF in Critically Ill Patients: A Systematic Review and Meta-Analysis

**DOI:** 10.3389/fmed.2021.705960

**Published:** 2021-08-10

**Authors:** Xueyan Yuan, Ling Liu, Wei Chang, Zongsheng Wu, Lili Huang, Yali Chao, Xinxing Lu, Jianfeng Xie, Yi Yang, Haibo Qiu

**Affiliations:** Jiangsu Provincial Key Laboratory of Critical Care Medicine, Department of Critical Care Medicine, School of Medicine, Zhongda Hospital, Southeast University, Nanjing, China

**Keywords:** lung ultrasound, diagnostic accuracy, consolidation, acute interstitial syndrome, acute respiratory failure

## Abstract

**Background:** Acute respiratory failure (ARF) is a commonly distressing condition in critically ill patients. Its early recognition and treatment may improve clinical outcomes. Mounting evidence suggests that lung ultrasound (LUS) could be an alternative to chest X-ray (CXR) or computed tomography (CT) for the diagnosis of ARF in critically ill patients. This meta-analysis aimed to determine whether LUS can be an alternative tool used to investigate the cause of ARF or thoracic pathologies associated with the diagnosis of ARF in critically ill patients.

**Method:** A systematic literature search of the PubMed, Web of Science, Embase, and Cochrane Library databases was conducted from inception to March 2020. Two researchers independently screened studies investigating the accuracy of LUS with CXR or CT for adult critically ill patients with ARF. Data with baseline, true positives, false positives, false negatives, and true negatives were extracted. The study quality was assessed using the Quality Assessment of Diagnostic Accuracy Studies-2 tool. The pooled sensitivity and specificity were obtained using a bivariate model.

**Results:** Eleven studies, including 1,232 patients, were included in the meta-analysis. Most studies were of low quality. LUS had a pooled sensitivity of 92% (95% confidence interval [CI]: 85–96) and a pooled specificity of 98% (95% CI: 94–99). The area under the summary receiver operating characteristic curve was 98% (95% CI: 97–99). The sensitivity and specificity of LUS to identify different pathological types of ARF were investigated. For consolidation (1,040 patients), LUS had a sensitivity of 89% and a specificity of 97%. For pleural effusion (279 patients), LUS had a pooled sensitivity of 95% and a specificity of 99%. For acute interstitial syndrome (174 patients), LUS had a pooled sensitivity of 95% and a specificity of 91%.

**Conclusions:** LUS is an adjuvant tool that has a moderate sensitivity and high specificity for the diagnosis of ARF in critically ill patients.

**Systematic Review Registration:** The study protocol was registered with PROSPERO (CRD42020211493).

## Introduction

Acute respiratory failure (ARF) is a commonly distressing condition in critically ill patients with increased incidence (1). The common causes of ARF include pneumonia, sepsis, heart failure, and chronic obstructive pulmonary disease (COPD) (1, 2). In most cases, patients with ARF present in conditions not suitable for the establishment of accurate diagnosis at the early stage of illness, which may compromise the outcomes (3, 4). Early recognition and treatment of ARF may play an important role in improving clinical outcomes.

The diagnosis of the underlying causes of ARF is dependent on chest imaging, with unreliable results. Chest X-ray (CXR) is recommended as the first-line imaging modality for the diagnosis of ARF in intensive care units (ICUs), but the limited supine films result in diminished diagnostic accuracy for consolidation (5). The “gold standard” modality, chest computed tomography (CT), has considerable limitation, although it currently offers a higher accurate diagnosis for lung lesions (6). This is related to the difficulties and risks of transportation, radiation exposure, and costs (7).

Lung ultrasound (LUS) is possibly a reliable diagnostic approach that can be used in critically ill patients (8). Studies have shown that the diagnostic accuracy of LUS for pneumonia was well-established (9–11). Available data have also suggested that LUS had a high diagnostic performance for commonly encountered conditions, such as pulmonary embolism and pneumothorax (12, 13). Furthermore, LUS is portable, inexpensive, radiation-free, non-invasive, and real-time at the bedside. Thus, LUS may be a potential alternative to chest radiography or CT for the diagnosis of ARF.

This systematic literature review and meta-analysis aimed to assess the diagnostic performance (including sensitivity and specificity) of LUS for the different pathological types of critically ill patients with ARF.

## Methods

We conducted this systematic review and meta-analysis to assess the diagnostic efficacy of LUS in ARF in accordance with the Preferred Reporting Items for Systematic Reviews and Meta-analyses (PRISMA) guidelines for Diagnostic Test Accuracy (14). The meta-analysis was prospectively registered at https://www.crd.york.ac.uk/prospero/ with the registration number CRD42020211493.

### Search Strategy and Study Selection

Two researchers (XY and LH) independently conducted an electronic database search, including PubMed, Embase, the Cochrane Library, and Web of Science databases, to identify potentially eligible studies published from inception to March 2020. The search strategy included controlled vocabulary (i.e., Medical Subject Headings) and free-text words for two basic concepts: (1) ultrasonography and (2) ARF, respiratory insufficiency, and ventilatory depression. The search strategy details are presented in Supplementary Table 1. Two researchers (XY and ZW) selected and evaluated the titles and abstracts of the retrieved literature. All disagreements between the two researchers were resolved by the intervention of a third expert (WC).

This systematic review and meta-analysis included all English-language articles describing retrospective and prospective observational studies. Studies were included if they (i) enrolled adult patients with clinically suspected or confirmed ARF caused by any etiology, (ii) compared the diagnostic accuracy of LUS for ARF with radiography or CT, and (iii) included more than 20 consecutive patients. The following studies were excluded: case reports, studies with abstracts without full text available, animal studies, and pediatric studies. The outcomes were all data concerning diagnostic accuracy including sensitivity, specificity, pooled positive likelihood ratio (PLR), pooled negative likelihood ratio (NLR), and diagnostic odds ratio (DOR) with 95% confidence intervals (CIs). PLR and NLR indicate the reliability of the results. The higher the PLR value, the greater the probability of the diagnosis of ARF with LUS. Meanwhile, NLR has a contrasting concept. A higher DOR indicates a higher diagnostic accuracy.

### Data Extraction

Two researchers (XL and YC) independently extracted the data including the number of true positives, false positives, false negatives, and true negatives with prepared data extraction forms. When the information we needed was not explicitly obtained in the selected studies, a 2 × 2 table was built to calculate the required data. Additionally, other data, including the year of the studies, settings, origins of patients, sample sizes, causes of ARF, ultrasound equipment, lung areas examined, and expertise of operators, were obtained.

### Quality Assessment

The Quality Assessment of Diagnostic Accuracy Studies-2 tool (QUADAS-2) was performed to assess the methodological quality of the selected studies (15). Studies with potential risk of bias for any domains were identified to have high risk of bias overall. Overall quality was independently determined by two researchers (XY and YC) with discrepancies solved by consensus.

### Data Analysis and Synthesis

The statistical analysis was performed in the raw data according to the European Association for Technology Assessment recommendations (16). The quality of the included studies was assessed using QUADAS-2 with Review Manager 5.3. The pooled sensitivity and specificity were obtained using a bivariate model with Stata 15.0. The Midas module included in the Stata statistical package was used to construct forest plots. Heterogeneity was estimated using the *Q*-test and the *I*^2^ statistic, and significant heterogeneity was considered when the *P*-value was < 0.05 or *I*^2^ was >50%. The summary receiver operating characteristic (SROC) curves were plotted to estimate the true positivity and specificity. Meanwhile, Fagan's nomogram and likelihood ratio plot were performed to assess the clinical applicability of LUS in diagnosing ARF. The causes of heterogeneity in the studies were identified using subgroup analysis and meta-regression analysis. A sensitivity analysis was performed to assess the stability of the results. Publication bias was estimated with Deeks' funnel plot asymmetry test, and significant publication bias was considered when the *P*-value was < 0.10.

## Results

### Literature Search

**Figure 1:**
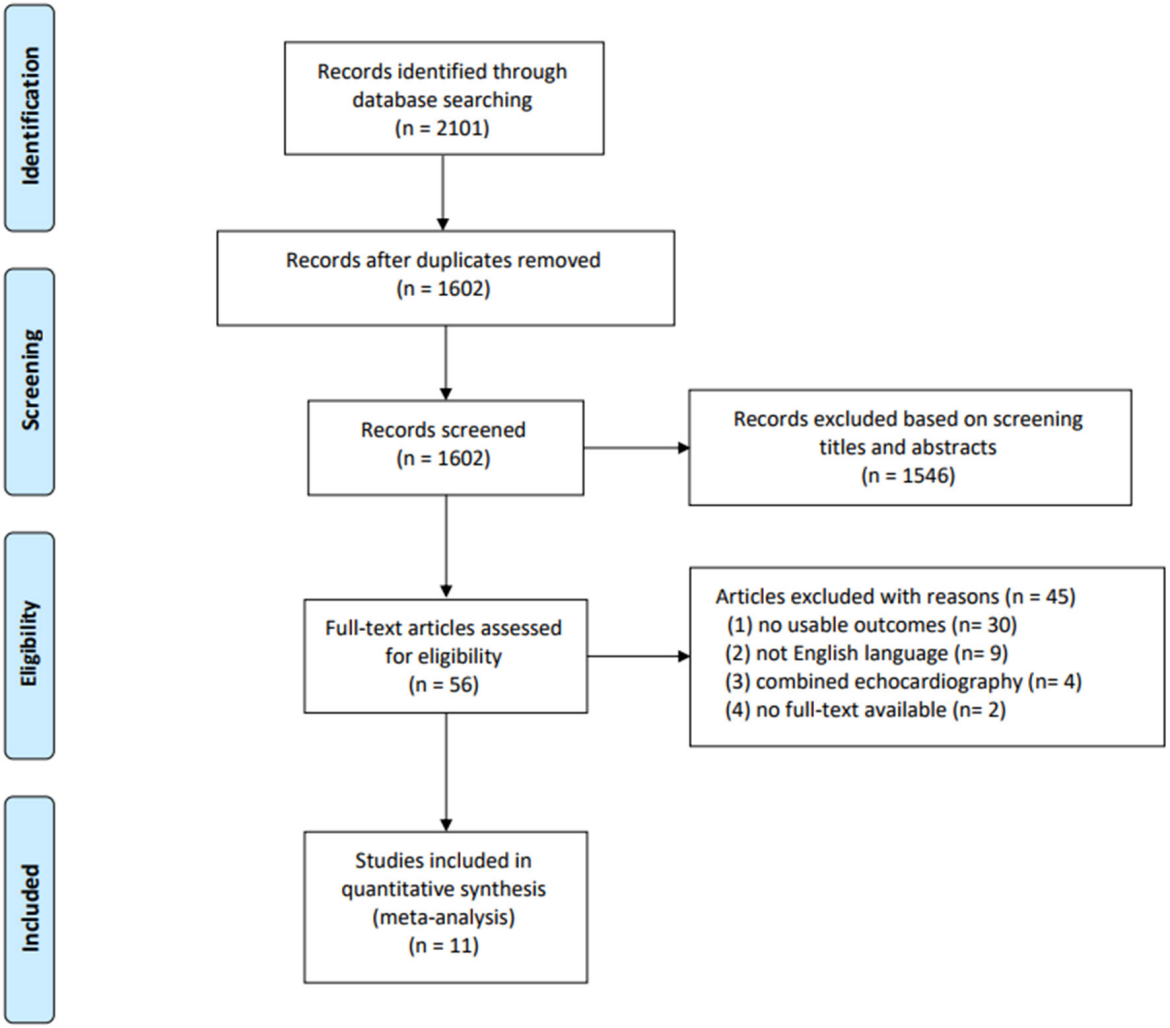
Flowchart of the selection process for the included studies.

Of the 2,102 studies obtained through the databases and references, 2,046 were excluded by screening the titles and abstracts. A total of 56 potentially eligible studies remained, of which 11 studies (1,232 patients) were finally included in the quantitative analyses (17–27). The details of the study selection and reasons for excluding studies are presented in Figure 1.

### Study Characteristics and Quality Assessment

All characteristics of the included studies are summarized in Table 1. All the included studies were conducted between 2004 and 2019. Ten studies, comprising 776 patients, had a prospective design (17–25, 27). Of the 11 included studies, five evaluated the diagnostic accuracy of LUS compared with CT (18, 21, 23, 24, 27). It is unclear whether the ultrasound operators were blinded to the results of chest radiography or CT in two studies (19, 21). Details of the performance of LUS are summarized in Supplementary Table 2.

**Table 1:** Characteristics of the included studies and patients.

**Study**	**Year**	**Setting**	**Country**	**Design**	**Number of patients and lung regions in the LUS protocol**	**Cause of ARF**	**Reference standard**	**Blind in reference standard**
Lichtenstein et al. (17)	2004	Surgical ICU	France	Prospective	ARDS (*n* = 32) and healthy controls (*n* = 10), total lung regions (*n* = 384)	ARDS (27 insulted to the lung, 5 insulted to secondary reason)	CXR	Yes
Lichtenstein et al. (18)	2004	Medical ICU	France	Prospective	ARF (*n* = 60), total lung regions (*n* = 118)	–	CT	Yes
Copetti et al. (19)	2008	ICU	Italy	Prospective	ARDS (*n* = 18) and APE (*n* = 40), total hemi-thoraces (*n* = 10)	ARDS (4 insulted to the lung, 11 insulted to secondary reason)	CXR	Unclear
Lichtenstein et al. (20)	2008	ICU	France	Prospective	ARF (*n* = 260), lung regions were unknown	COPD, cardiogenic pulmonary edema, pneumonia, acute asthma, pulmonary embolism, pneumothorax	CT/CXR	Yes
Rocco et al. (21)	2008	Mixed ICU	Italy	Prospective	Trauma, requiring mechanical ventilation (*n* = 22): total lung regions (*n* = 180)	Thoracic trauma	CT	Unclear
Xirouchaki et al. (22)	2011	Mixed ICU	Greece	Prospective	Mechanical ventilation (*n* = 42): total of hemi-thoraces (*n* = 84)	–	CXR	Yes
Refaat and Abdurrahman (23)	2013	Chest ICU	Egypt	Prospective	ARF (*n* = 90), lung regions were unknown	–	CT	Yes
Daabis et al. (24)	2014	ICU	Egypt	Prospective	ARF (*n* = 93), lung regions were unknown	–	CT	Yes
Bass et al. (25)	2015	Mixed ICU	USA	Prospective	Mechanical ventilation (*n* = 77), lung regions were unknown	–	CXR	Yes
See et al. (26)	2018	ICU	Singapore	Retrospective	ARDS (*n* = 216) and non-ARDS (*n* = 240), total hemi-thoraces (*n* = 12)	ARDS (100 insulted to lung, 356 insulted to the secondary reason)	CXR	Yes
Chiumello et al. (27)	2019	ICU	Italy	Prospective	ARDS (*n* = 32); total hemi-thoraces (*n* = 12)	ARDS (25 insulted to the lung, 7 insulted to secondary reason)	CT	Yes

*LUS, lung ultrasound; ARF, acute respiratory failure; ARDS, acute respiratory distress; CXR, chest X-ray; CT, computed tomography*.

**Figure 2:**
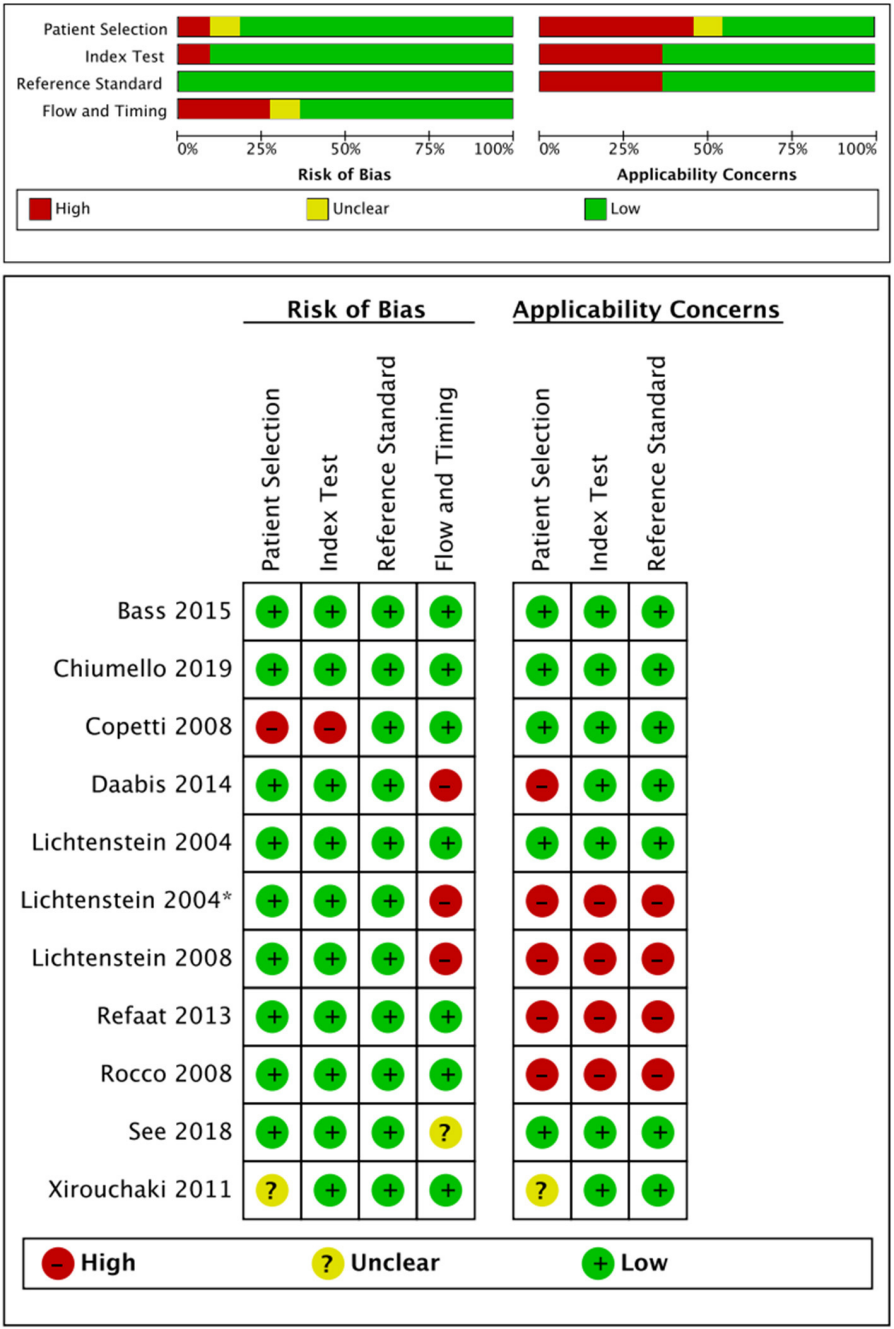
Risk of bias and applicability concerns assessment according to the QUADAS-2. QUADAS-2, Quality Assessment of Diagnostic Accuracy Studies Score-2.

The quality assessment is presented in Figure 2. Most studies were of low quality according to the QUADAS-2 criteria. Concerning patient selection, some studies were at high risk of bias and compromised the applicability (18, 20, 24). One study was at high risk of bias but did not compromise the applicability in an index test (19). Considering the flow and timing, one study was at high risk of bias but did not compromise the applicability (19).

### Diagnostic Accuracy of LUS in Critically Ill Patients With ARF

**Figure 3:**
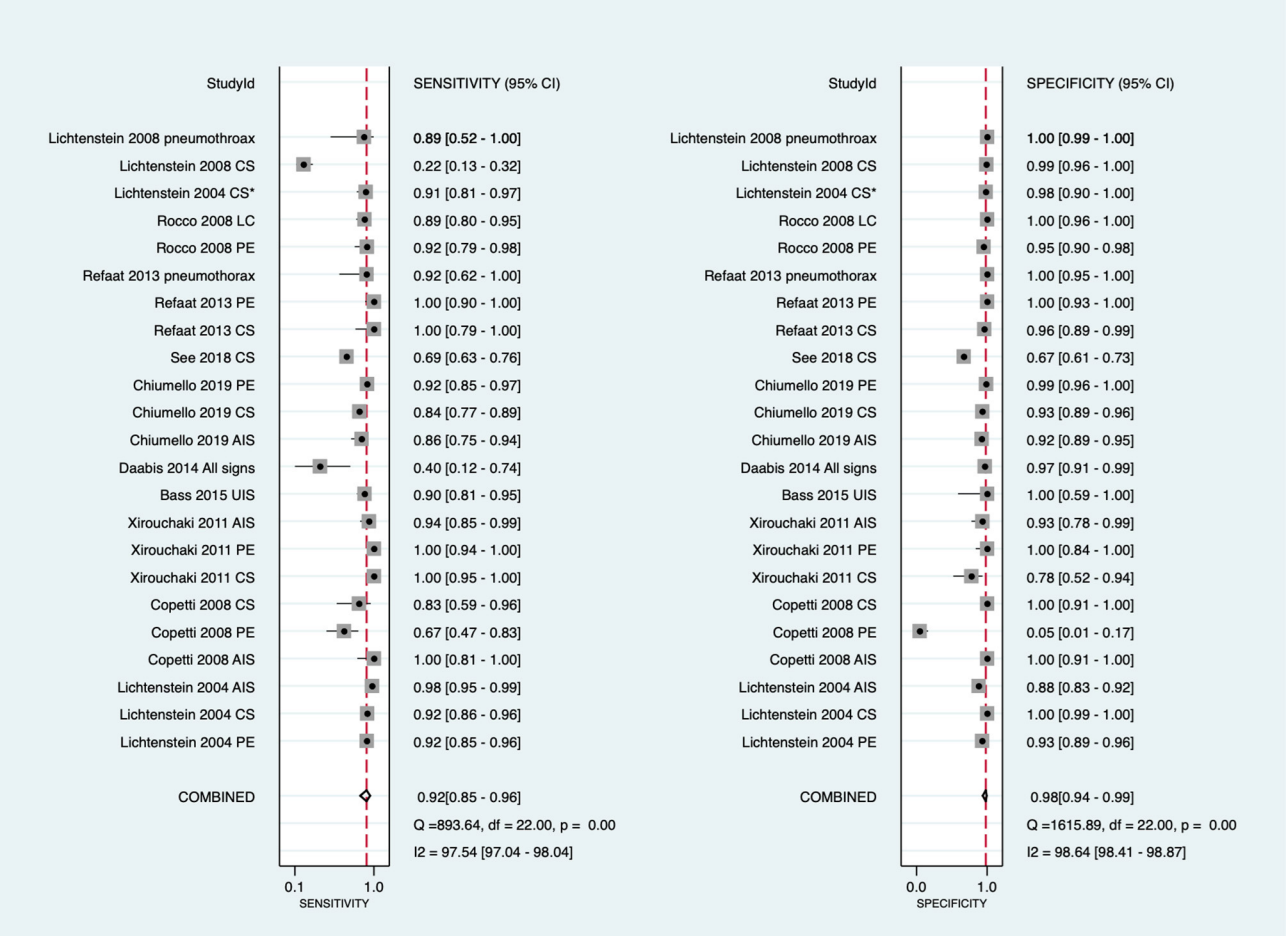
Forest plot of the sensitivity and specificity in the overall studies. Several studies used repeated measurements per patient (e.g., measurements on different types of pathology episodes and different lung fields). *Daabis et al. (24) included all profiles including A, AB, B, B+PLAPS, and lung point. CI, confidence interval; LUS, lung ultrasound; CS, consolidation; PE, pleural effusion; LC, lung contusion; AIS, acute interstitial syndrome; B, B lines; PLAPS, post-erolateral alveolar and/or pleural syndrome; UIS, ultrasound interstitial syndrome.

**Figure 4:**
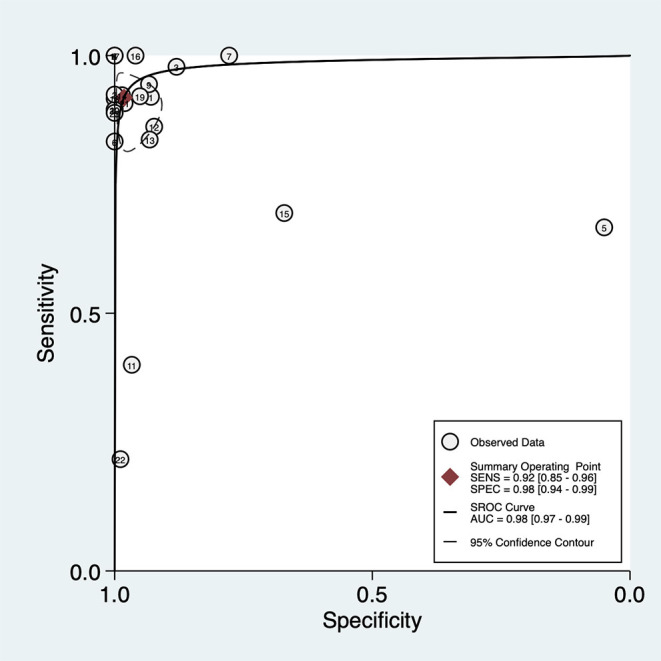
Summary of the receiver operating characteristic curve (SROC) of lung ultrasound in critically ill patients with acute respiratory failure. AUC, area under curve.

The overall pooled sensitivity and specificity of LUS were 92% (95% CI: 85–96) and 98% (95% CI: 94–99), respectively (Figure 3). In addition, the overall area under the SROC curve (AUC) of LUS was 98% (95% CI: 97–99), indicating that LUS had a high diagnostic value for ARF (Figure 4). The sensitivity, specificity, PLR, NLR, and DOR of different types of pathology were investigated, and the main results are presented in Table 2. For consolidation, eight studies, comprising 1,040 patients, were included (17–20, 22, 23, 26, 27). The pooled sensitivity and specificity of LUS were 89 and 97%, respectively. The PLR, NLR, and DOR were 31.9, 0.11, and 284, respectively. Six studies, comprising 279 patients, reported the raw data for pleural effusion (17, 19, 21–23, 27). LUS had a pooled sensitivity of 95% and a specificity of 99%. The PLR, NLR, and DOR were 88.1, 0.05, and 1,750, respectively. Four studies, comprising 174 patients, reported the accuracy of LUS to identify acute interstitial syndrome (AIS) (17, 19, 22, 27). The sensitivity and the specificity were 95 and 91%, respectively. Furthermore, the PLR, NLR, and DOR were 10.8, 0.06, and 196, respectively. Pneumothorax was investigated in two studies (20, 23). The pooled sensitivity and specificity for pneumothorax of LUS were 90 and 100%, respectively. Lung contusion was examined in one study (21), which reported an LUS sensitivity and specificity of both 89%. The SROC curves for different types of pathology are presented in Supplementary Figure 1.

**Table 2:** Diagnostic performance of lung ultrasound in critically ill patients with acute respiratory failure.

	**Consolidation**	**AIS**	**PE**	**Pneumothorax**
No. of studies (patients)	8 (1,040)	4 (174)	6 (279)	3 (332)
SEN (95% CI) (%)	89 (66–97)	95 (88–98)	95 (82–99)	90 (70–99)
SPE (95% CI) (%)	97 (88–99)	91 (88–94)	99 (47–100)	100 (0.99–100)
PLR (95% CI)	31.9 (7.4–137.2)	10.8 (8.1–14.6)	88.1 (0.8–9193.2)	244.2 (34.3–1737.9)
NLR (95% CI)	0.11 (0.03–0.41)	0.06 (0.02–0.13)	0.05 (0.01–0.21)	0.13 (0.05–0.38)
DOR (95% CI)	284 (41–1,970)	196 (85–453)	1750 (4–6,93,798)	1849 (184–18,622)

*PE, pleural effusion; AIS, acute interstitial syndrome; DOR, diagnostic odds ratio; PLR, positive likelihood ratio; NLR, negative likelihood ratio; CI, confidence interval*.

### Causes of Heterogeneity

The significant heterogeneity in the meta-analysis was performed according to the dispersion of studies in the ROC plane. An influence analysis was performed to examine the potential sources of heterogeneity in Supplementary Figure 2. The results suggested that there was no outlier for consolidation, PE, and AIS. To further explore the heterogeneity of the included studies, the secondary analysis according to different reference standards (CT or CXR) was performed (Table 3). With CT as the reference standard, the sensitivity and specificity of LUS for the diagnosis of consolidation were 86 and 95%. With CXR as the reference standard, LUS had sensitivities of 73 and 97% and specificities of 99 and 90% to identify consolidation and AIS, respectively.

**Table 3:** Summary of the performance of different reference standards for the diagnosis of ARF in critically ill patients.

	**No. of studies (patients)**	**SEN (%)**	**SPE (%)**	**PLR**	**NLR**	**DOR**
		**(95% CI)**	**(95% CI)**	**(95% CI)**	**(95% CI)**	**(95% CI)**
**REFERENCE STANDARD: CT**						
Consolidation	3 (182)	86 (82–90)	95 (92–97)	15.5 (8.9–26.9)	0.13 (0.07–0.24)	192 (37–980)
**REFERENCE STANDARD: CXR**						
Consolidation	4 (598)	73 (68–78)	99 (97–100)	32.4 (3.0–353.1)	0.12 (0.01–2.29)	330 (23–4725)
AIS	3 (142)	97 (94–99)	90 (86–94)	11.6 (4.8–27.7)	0.04 (0.02–0.07)	342 (138–848)

*PE, pleural effusion; AIS, acute interstitial syndrome; DOR, diagnostic odds ratio; PLR, positive likelihood ratio; NLR, negative likelihood ratio; CI, confidence interval; CT, computed tomography; CXR, chest X-ray*.

### Assessment of Clinical Applicability

Fagan's nomogram on the pre-test and post-test probability of LUS for diagnosing ARF in critically ill patients is shown in Supplementary Figure 3. Fagan's nomogram showed that the pooled PLR and NLR were 51 and 0.08, respectively.

### Publication Bias

**Figure 5:**
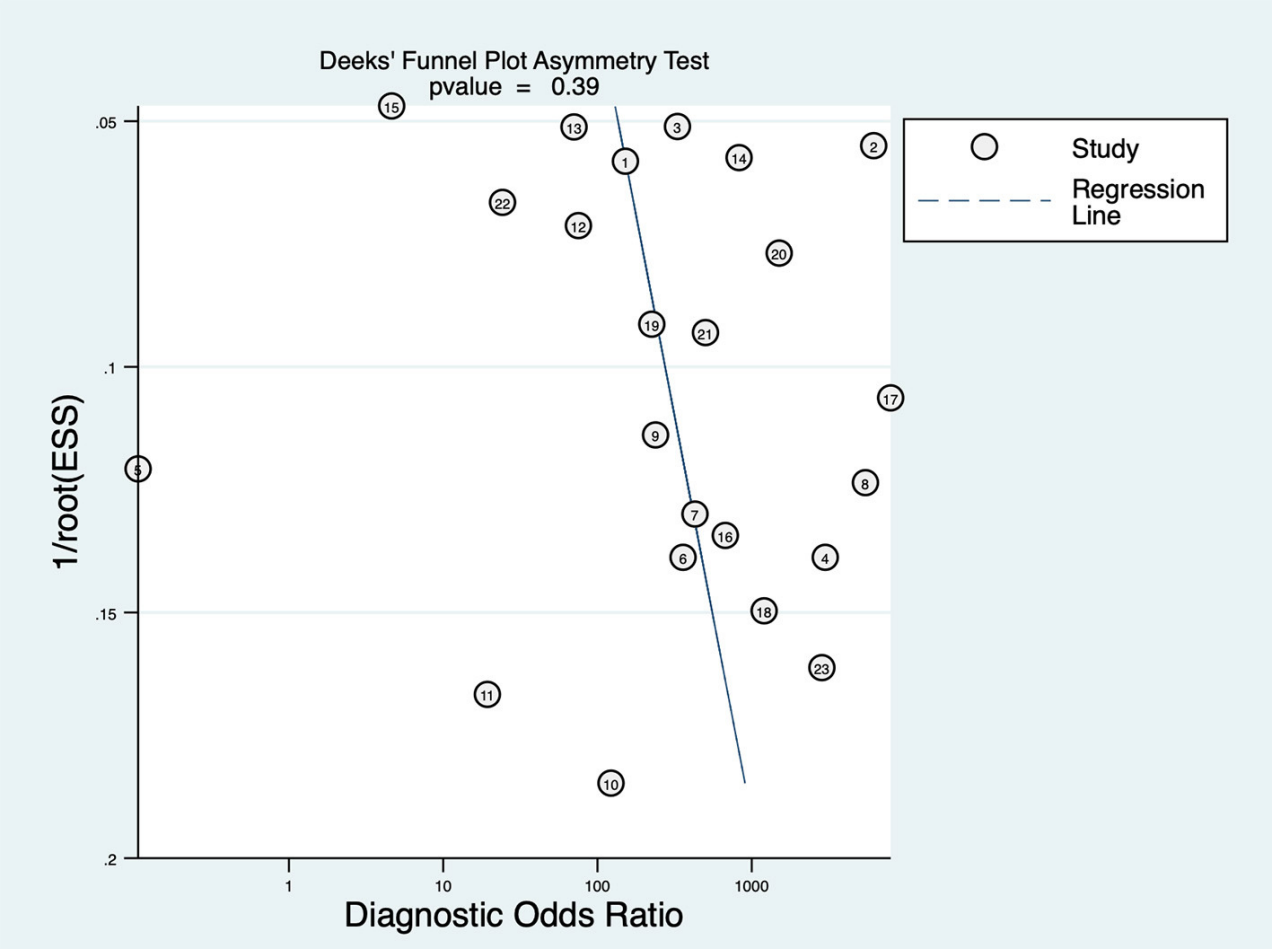
Graph of Deeks' funnel plot asymmetry.

Deeks' funnel plot asymmetry test was performed to examine publication bias (Figure 5), which showed that there was no significant publication bias in this study (*P* = 0.39).

## Discussion

In this systematic review and meta-analysis, 11 studies were included, and the diagnostic value of LUS was investigated. The results indicated that LUS had an overall moderate sensitivity of 92% (95% CI: 85–96) and high specificity of 98% (95% CI: 94–99) for diagnosing of ARF in critically ill patients. The secondary analysis was performed to determine the significant heterogeneity, and the result showed that LUS had a low sensitivity but high specificity in diagnosing consolidation regardless of whether CXR or CT was used as the reference standard. LUS had a high sensitivity but low specificity to identify AIS in ARF with CXR as the reference standard.

LUS, as a convenient approach, has routinely been used in critically ill patients and has been identified to be effective in evaluating ICU conditions such as pneumonia (9) and pneumothorax (13). Despite the pervasive use of LUS in critically ill patients, few studies have focused on the diagnostic value of LUS in ARF. This meta-analysis demonstrates that LUS had a moderate sensitivity and high specificity to identify ARF when compared with CXR or CT. The results are relatively different from those of a recent meta-analysis in which the pooled sensitivity and specificity for the diagnostic accuracy of LUS in critically ill patients with respiratory symptoms were 95 and 94%, respectively (28). In this study, the authors only focused on the diagnostic accuracy of LUS with CT as the reference standard. Staub et al. have also explored the diagnostic value of LUS in adults with respiratory symptoms (29). However, they mainly focused on patients with pneumonia, acute heart failure, and exacerbations of COPD in the emergency department. They reported that LUS had sensitivity of ~85–95% and specificity of 75–90%. This result is possibly attributed to the heterogeneity of the population included in these studies.

Common pathological types, including consolidation, AIS, and PE in ARF, were the main concern in the present study. LUS in ARF with PE had higher sensitivity and specificity than consolidation and AIS. LUS was low sensitive but highly specific for the diagnosis of consolidation in critically ill patients with ARF. However, although AIS is central to the BLUE protocol (30), LUS had a high sensitivity but low specificity to identify AIS in this study. This may be explained by the fact that LUS can detect the interstitial edema surrounding an isolated consolidation, even if deep consolidations are not detected (31). These results are consistent with the results of a recent study. Chinardet et al. (32) have demonstrated that LUS was useful in evaluating consolidation after PE drainage in acute respiratory distress syndrome (ARDS). However, there were only 10 patients in this study.

Due to the rapid development of ARF, early changes in lung morphology can occur, which can be assessed by LUS. This review identified that LUS might be a valuable resource as an adjunct or replacement for CXR and CT in critically ill patients with ARF in clinical practice. Compared with CXR and CT, LUS has some advantages in addition to its diagnostic accuracy. LUS is convenient and can be performed right at the bedside (33, 34). Furthermore, LUS is inexpensive and can be used worldwide, especially in areas where medical sources are limited. Considering these advantages, LUS is considered a routine imaging modality for critically ill patients, especially for patients with unstable conditions. LUS is simply performed, but it must be methodologically learned. The main disadvantage of LUS that restricts its use is it is highly operator dependent (9). The heterogeneity of the observation-dependent nature of LUS may affect the reliability of the study results. Furthermore, similar with CXR and CT, the images obtained in LUS are only considered useful when these are combined with clinical information. Hence, combined with clinical information, well-trained operators select LUS as their preferred choice of modality.

Our results revealed substantial heterogeneity in the included studies, and the reasons for heterogeneity were investigated by subgroup analysis. First, the study comprised several different pathologies, including consolidation, AIS, PE, LC, and pneumothorax. This may affect the diagnostic accuracy because different pathologies have different values in diagnosing ARF. To reduce heterogeneity, we further investigated the sensitivity and specificity of the main pathologies, including consolidation, AIS, PE, and pneumothorax. The heterogeneity was subject to these aspects in pioneering meta-analysis on the diagnosis of LUS (10, 11). Second, several included studies were of low quality. The different study qualities can lead to heterogeneity. A previous study by Llamas-Álvarez et al. (9) has attributed the heterogeneity to the study quality, and the diagnostic accuracy of LUS improved by stratifying the study quality.

This meta-analysis has several limitations. First, this meta-analysis included a limited number of studies, with only two studies involving 100 participants or more (20, 26). This may lead to the non-repeatability of the results, and the results need to be interpreted carefully. Second, only four different pathologies were investigated for heterogeneity in our study. Although we performed subgroup analysis, the heterogeneity could not be fully explained because there were more manifestations of these four pathologies in LUS. Third, the study quality was limited by the included literature due to the secondary analysis.

## Conclusions

This systematic review and meta-analysis demonstrated that LUS had moderate sensitivity and high specificity for diagnosing ARF in critically ill patients when compared with CXR or CT. LUS seems to be a well-validated modality to investigate the cause of ARF or thoracic pathology associated with the diagnosis. However, large-scale studies are needed to confirm the role of LUS in critically ill patients with ARF.

## Data Availability Statement

The original contributions presented in the study are included in the article/Supplementary Material, further inquiries can be directed to the corresponding author/s.

## Author Contributions

HQ and XY conceptualized and designed the study, collected and organized the data, and drafted the initial manuscript. LH, ZW, YC, and XL collected and organized the data, reviewed the included articles, and conducted the analyses. WC collected and organized the data and reviewed the included articles. LL and JX conceptualized and designed the study, and critically reviewed and revised the manuscript. YY conceptualized and designed the study, coordinated and supervised data collection, and critically reviewed and revised the manuscript. All authors read and approved the final manuscript.

## Conflict of Interest

The authors declare that the research was conducted in the absence of any commercial or financial relationships that could be construed as a potential conflict of interest.

## Publisher's Note

All claims expressed in this article are solely those of the authors and do not necessarily represent those of their affiliated organizations, or those of the publisher, the editors and the reviewers. Any product that may be evaluated in this article, or claim that may be made by its manufacturer, is not guaranteed or endorsed by the publisher.
