# Supplementary material for: Diagnosis Accuracy of Lung Ultrasound for ARF in Critically Ill Patients: A Systematic Review and Meta-Analysis
Source: Front Med (Lausanne). 2021 Aug 10;8:705960. doi: 10.3389/fmed.2021.705960 (PMC8383297; doi:10.3389/fmed.2021.705960)

**Supplementary file**

**Supplementary Table 1.** The search strategy details

**PubMed:** #1 (((((((((((((((((((((((((Ultrasonography[Title/Abstract]) OR (Diagnostic Ultrasound[Title/Abstract])) OR (Diagnostic Ultrasounds[Title/Abstract])) OR (Ultrasound, Diagnostic[Title/Abstract])) OR (Ultrasounds, Diagnostic[Title/Abstract])) OR (Ultrasound Imaging[Title/Abstract])) OR (Imaging, Ultrasound[Title/Abstract])) OR (Imagings, Ultrasound[Title/Abstract])) OR (Echotomography[Title/Abstract])) OR (Ultrasonic Imaging[Title/Abstract])) OR (Imaging, Ultrasonic[Title/Abstract])) OR (Sonography, Medical[Title/Abstract])) OR (Medical Sonography[Title/Abstract])) OR (Ultrasonographic Imaging[Title/Abstract])) OR (Imaging, Ultrasonographic[Title/Abstract])) OR (Imagings, Ultrasonographic[Title/Abstract])) OR (Ultrasonographic Imagings[Title/Abstract])) OR (Echography[Title/Abstract])) OR (Diagnosis, Ultrasonic[Title/Abstract])) OR (Diagnoses, Ultrasonic[Title/Abstract])) OR (Ultrasonic Diagnoses[Title/Abstract])) OR (Ultrasonic Diagnosis[Title/Abstract])) OR (Echotomography, Computer[Title/Abstract])) OR (Computer Echotomography[Title/Abstract])) OR (Tomography, Ultrasonic[Title/Abstract])) OR (Ultrasonic Tomography[Title/Abstract])

#2 ((((((((((((((((((Respiratory Insufficiency[Title/Abstract]) OR (Acute Hypercapnic Respiratory Failure[Title/Abstract])) OR (Hypercapnic Acute Respiratory Failure[Title/Abstract])) OR (Acute Hypoxemic Respiratory Failure[Title/Abstract])) OR (Hypoxemic Acute Respiratory Failure[Title/Abstract])) OR (Respiratory Failure[Title/Abstract])) OR (Failure, Respiratory[Title/Abstract])) OR (Respiratory Failures[Title/Abstract])) OR (Hypoxemic Respiratory Failure[Title/Abstract])) OR (Failure, Hypoxemic Respiratory[Title/Abstract])) OR (Hypoxemic Respiratory Failures[Title/Abstract])) OR (Respiratory Failure, Hypoxemic[Title/Abstract])) OR (Hypercapnic Respiratory Failure[Title/Abstract])) OR (Failure, Hypercapnic Respiratory[Title/Abstract])) OR (Hypercapnic Respiratory Failures[Title/Abstract])) OR (Respiratory Failure, Hypercapnic[Title/Abstract])) OR (Respiratory Depression[Title/Abstract])) OR (Ventilatory Depression[Title/Abstract])) OR (Depressions, Ventilatory[Title/Abstract])

#1 AND #2

Total 487

**Embase**

#1 'ultrasonography' OR 'ultrasonic':ab,ti OR 'ultrasonics':ab,ti OR 'ultrasound':ab,ti OR 'ultrasounds':ab,ti OR 'ultra sound':ab,ti OR 'ultra sounds':ab,ti OR 'ultra- shell':ab,ti OR 'ultra shell':ab,ti OR 'lus':ab,ti OR 'sonography':ab,ti OR 'sonographies':ab,ti OR 'sonofication':ab,ti OR 'ultrasonography':ab,ti OR 'ultrasonographies':ab,ti OR 'echography':ab,ti OR 'echographies':ab,ti OR 'sonogram':ab,ti OR 'sonograms':ab,ti OR 'echogram':ab,ti OR 'echograms':ab,ti OR 'echoscopy':ab,ti OR 'echoscopies':ab,ti OR 'lung ultrasound':ab,ti OR 'chest ultrasound':ab,ti OR 'thoracic ultrasound':ab,ti OR 'lung ultrasounds':ab,ti OR 'chest ultrasounds':ab,ti OR 'thoracic ultrasounds':ab,ti OR 'lung ultrasonography':ab,ti OR 'lung ultrasonographies':ab,ti OR 'chest ultrasonography':ab,ti OR 'chest ultrasonographies':ab,ti OR 'thoracic ultrasonography':ab,ti OR 'thoracic ultrasonographies':ab,ti OR 'lung sonography':ab,ti OR 'lung sonographies':ab,ti OR 'chest sonography':ab,ti OR 'chest sonographies':ab,ti OR 'thoracic sonography':ab,ti OR 'thoracic sonographies':ab,ti OR 'lung echoschopy':ab,ti OR 'lung echoscopies':ab,ti OR 'chest echoscopy':ab,ti OR 'chest echoscopies':ab,ti OR 'thoracic echoschopy':ab,ti OR 'thoracic echoschopies':ab,ti OR 'lung echogram':ab,ti OR 'lung echograms':ab,ti OR 'lung sonogram':ab,ti OR 'lung sonograms':ab,ti OR 'chest sonogram':ab,ti OR 'chest sonograms':ab,ti OR 'thoracic sonogram':ab,ti OR 'thoracic sonograms':ab,ti OR 'lung ultra sound':ab,ti OR 'chest ultra sound':ab,ti OR 'thoracic ultra sound':ab,ti

#2 'respiratory insufficiency'/exp/mj OR 'acute hypercapnic respiratory failure':ab,ti OR 'hypercapnic acute respiratory failure':ab,ti OR 'acute hypoxemic respiratory failure':ab,ti OR 'hypoxemic acute respiratory failure':ab,ti OR 'respiratory failure':ab,ti OR 'failure, respiratory':ab,ti OR 'respiratory failures':ab,ti OR 'hypoxemic respiratory failure':ab,ti OR 'failure, hypoxemic respiratory':ab,ti OR 'hypoxemic respiratory failures':ab,ti OR 'respiratory failure, hypoxemic':ab,ti OR 'hypercapnic respiratory failure':ab,ti OR 'failure, hypercapnic respiratory':ab,ti OR 'hypercapnic respiratory failures':ab,ti OR 'respiratory failure, hypercapnic':ab,ti OR 'respiratory depression':ab,ti OR 'ventilatory depression':ab,ti OR 'depressions, ventilatory':ab,ti

#3 #1 AND #2

Total 741

**Web of Science**

#1 TOPIC: (Respiratory Insufficiency) OR TOPIC: (acute hypercapnic respiratory failure) OR TOPIC: (acute hypoxemic respiratory failure) OR TOPIC: (respiratory failure) OR TOPIC: (respiratory failures) OR TOPIC: (hypoxemic respiratory failure) OR TOPIC: (hypoxemic respiratory failures) OR TOPIC: (hypercapnic respiratory failure) OR TOPIC: (hypercapnic respiratory failures) OR TOPIC: (respiratory depression) OR TOPIC: (ventilatory depression)

#2 TOPIC: (Ultrasonography) OR TOPIC: (Diagnostic Ultrasound) OR TOPIC: (Diagnostic Ultrasounds) OR TOPIC: (Ultrasound, Diagnostic) OR TOPIC: (Ultrasounds, Diagnostic) OR TOPIC: (Ultrasound Imaging) OR TOPIC: (Imaging, Ultrasound) OR TOPIC: (Imagings, Ultrasound) OR TOPIC: (Echotomography) OR TOPIC: (Ultrasonic Imaging) OR TOPIC: (Imaging, Ultrasonic) OR TOPIC: (Sonography, Medical) OR TOPIC: (Medical Sonography) OR TOPIC: (Ultrasonographic Imaging) OR TOPIC: (Imaging, Ultrasonographic) OR TOPIC: (Imagings, Ultrasonographic) OR TOPIC: (Ultrasonographic Imagings) OR TOPIC: (Echography) OR TOPIC: (Diagnosis, Ultrasonic) OR TOPIC: (Ultrasonic Tomography) OR TOPIC: (Ultrasonic Diagnoses) OR TOPIC: (Ultrasonic Diagnosis) OR TOPIC: (Tomography, Ultrasonic) OR TOPIC: (Computer Echotomography)

#3 (#2) AND #1 and ARTICLE OR EDITORIAL MATERIAL OR MEETING ABSTRACT OR PROCEEDINGS PAPER OR EARLY ACCESS OR LETTER OR NOTE (Document Types)

Total 713

**Cochrane**

#1 respiratory insufficiency

#2 acute hypercapnic respiratory failure

#3 acute hypoxemic respiratory failure

#4 respiratory failure

#5 respiratory failures

#6 hypoxemic respiratory failure

#7 hypoxemic respiratory failures

#8 hypercapnic respiratory failure

#9 hypercapnic respiratory failures

#10 respiratory depression

#11 ventilatory depression

#12 #1 or #2 or #3 or #4 or #5 or #6 or #7 or #8 or #9 or #10 or #11 in Trials

#13 Ultrasonography

#14 Diagnostic Ultrasound

#15 Diagnostic Ultrasounds

#16 Ultrasound Imaging

#17 Imagings, Ultrasound

#18 Echotomography

#19 Ultrasonic Imaging

#20 Sonography, Medical

#21 Ultrasonographic Imaging

#22 Echography

#23 Diagnosis, Ultrasonic

#24 Echotomography, Computer

#25 Tomography, Ultrasonic

#26 #13 or #14 or #15 or #16 or #17 or #18 or #19 or #20 or #21 or #22 or #23 or #24 or #25 in Trials

#27 #12 and #26

Total 216

**Supplementary Table 2.** Details of the performance of lung ultrasound

| Study | Ultrasound equipment | Lung areas examined/ Patient position | Diagnostic criteria | Operators’ characteristics |
| --- | --- | --- | --- | --- |
| Lichtenstein  et al ^16^ | Hitachi-405 (Hitachi Medical Corporation, Tokyo, Japan), a microconvex 5 MHz, 9-cm-long probe | 12 lung regions | LUS: AIS: >2 B lines in a given region. CS: air bronchogram. PE: a dependent collection limited by the diaphragm and the pleura; CXR: 12 regions, according to Fleischner society and lung pathology according to ARDS criteria. | A single operator |
| Lichtenstein  et al ^17^ | Hitachi Sumi 405 (Hitachi Medical Corporation, Tokyo, Japan) with a 3.5 MHz micro-convex probe | The thorax was distinguished from the abdomen by locating the diaphragm, usually at the mamillary line or one or two intercostal spaces below in a supine patient | LUS: CS: air bronchogram; CT: from the apex to the diaphragm. Alveolar CS: a tissular pattern visible at the mediastinal window | Two operators |
| Copetti  et al ^18^ | convex probe 3.5–5 MHz and linear probe 7.5–10 MHz (Megas CVX Esaote Medical Systems, Florence-Italy) | 10 regions (2 anterior, 2 lateral, 1 posterior) | LUS: AIS: > 3 ULCs or "white lung". CS: air bronchogram. PE: a dependent collection limited by the diaphragm and the pleura. Lung pulse: absence of lung sliding with the perception of heart activity at the pleural line; CXR: regions not reported, lung pathology according to ARDS criteria. | —— |
| Lichtenstein  et al ^19^ | Hitachi-405 (Hitachi Medical; Tokyo, Japan) with a 5-MHz microconvex probe | semirecumbent position, or supine if intubated | LUS: IS: >2 B lines in a given region. CS: tissular pattern, absence of the lung line, absence of the sinusoid sign. PE: anechoic-dependent pattern; CXR/CT: regions not reported, according to Fleischner society and lung pathology according to ARF criteria. | —— |
| Rocco  et al ^20^ | Aloka SSD 1700 (Aloka Co Ltd, Tokyo, Japan) and a convex 3.5 MHz, 9-cm-long probe. | 12 lung regions | LUS: PE: a dependent collection limited by the diaphragm and the pleura. LC, air bronchograms or >2 B lines and A lines; CXR: 12 regions; PE: a generalized increased in opacity of a hemithorax. LC: a homogeneous hazy parenchymal CS or an interstitial form of irregular coarse infiltration; CT: 12 regions; PE: the presence of liquid in pleural space. LC: either areas of homogeneous air-space consolidation or an interstitial form of irregular coarse infiltration. | Two trained operators |
| Xirouchaki  et al ^21^ | microconvex 5–9 MHz transducer appropriate for transthoracic examination (HITACHI EUB 8500) | six regions in each hemithorax, three in upper fields (anterior, posterior, lateral) and three in lower fields (anterior, posterior, lateral) | LUS: AIS: >2 B lines in a given region. CS: air bronchogram. PE: a dependent collection limited by the diaphragm and the pleura; CXR: 12 regions according to Fleischner society. | —— |
| Refaat  et al ^22^ | Linear 7.5–10 MHz and convex 3.5 MHz, Portable Shenzhen mindray DP-1100 Plus) | 12 lung regions | LUS: CS: hypoechoic pattern, non-homogenous echo-texture, irregular shape, serrated margin, air and fluid bronchograms. PE: anechoic space between visceral and parietal pleura, or parenchymal atelectasis. Pneumothorax: absent lung sliding in combination with the A-line sign; CT: obtained in the supine position from the apex of the thorax to the lung bases. | A single operator |
| Bass  et al ^24^ | Sonosite SICU ultrasound machine (SonoSite, Inc., Bothell, Washington) with p21x 5–1 Mhz phased array probe | six locations: a zone 1 is 2 cm below the anterior mid-clavicular line on the right side of the chest; b zone 2 is 4 cm inferior and 4 cm lateral to zone 1; c zone 3 is 2 cm inferior to zone 2 along the mid-axillary line. d–f the identical positions on the left side of the chest | LUS: UIS: >2 B lines in a single frozen frame in one or more lung fields; CXR: regions not reported, bilateral radiographic opacities not fully explained by PE, lobar/lung collapse, or nodules. | physician received 4 h of training |
| Daabis  et al ^23^ | Both deep (2.5 MHZ) and superficial (5 MHZ) probes | the regions not reported. Semirecumbent position, or supine if intubated | LUS: B profile, B + PLAPS profile; CXR: not reported. | a specialist |
| See  et al ^25^ | Sparq Ultrasound System (Philips Health- care, Andover, MA) equipped with a 2–4 MHz broadband sector phased array transducer | Six regions of each hemi-thorax (anterior-superior, anterior-inferior, lateral-superior, lateral-inferior, posterior-superior, and posterior-inferior) | LUS: B lines. CS: air bronchogram; CXR: regions not reported, bilateral radiographic opacities not fully explained by pleural effusions, lobar/lung collapse, or nodules. | trained respiratory therapists |
| Chiumello  et al ^26^ | Mindray TE7 Ultrasound System (Mindray Medical International, Shenzhen, China) with 2.5 MHz phased-array probe for tissue-like pattern and 12 MHz linear probe for pleural line | Each hemithorax was divided into anterior, lateral, and posterior region, according to landmarks set by the anterior and posterior axillary lines; each region was then divided in a superior and inferior half. Six areas per hemithorax were identified | LUS: AIS: >3 B-lines. CS: subpleural echo-poor region or one with tissue-like echotexture. PE: anechoic space between parietal and visceral pleura; CXR: 12 regions, AIS: ground-glass opacities, or a reticulation made of innumerable interlacing line shadows, or septal thickening. CS: attenuation obscuring margins of vessels and airway; PE: homogeneous and peripheral opacification. | one expert physician |

Abbreviations: AIS, acute interstitial syndrome; CS, consolidation; PE: pleural effusion; ARDS, acute respiratory distress syndrome; CXR: chest x-ray; CT, computed tomography; LC, lung contusions.

**Supplementary Figure 1.** The SROC curve for different types of pathology.

Abbreviations: SROC, Summary receiver operating characteristic


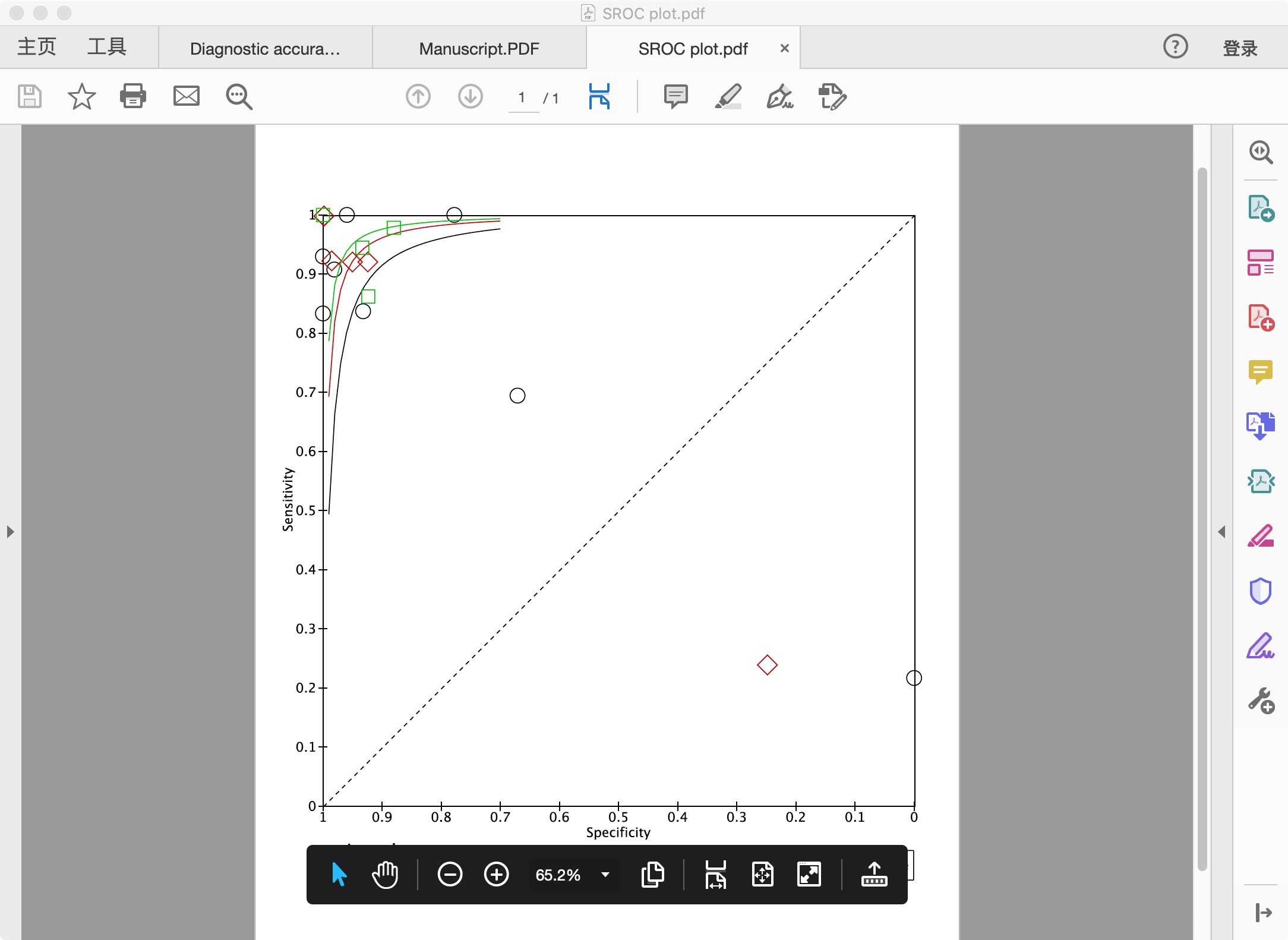


**Supplementary Figure 2.** The influence analysis for the heterogeneity in three different pathologies. a. Consolidation; b. PE; c. AIS

Abbreviations: PE, pleural effusion; AIS, acute interstitial syndrome


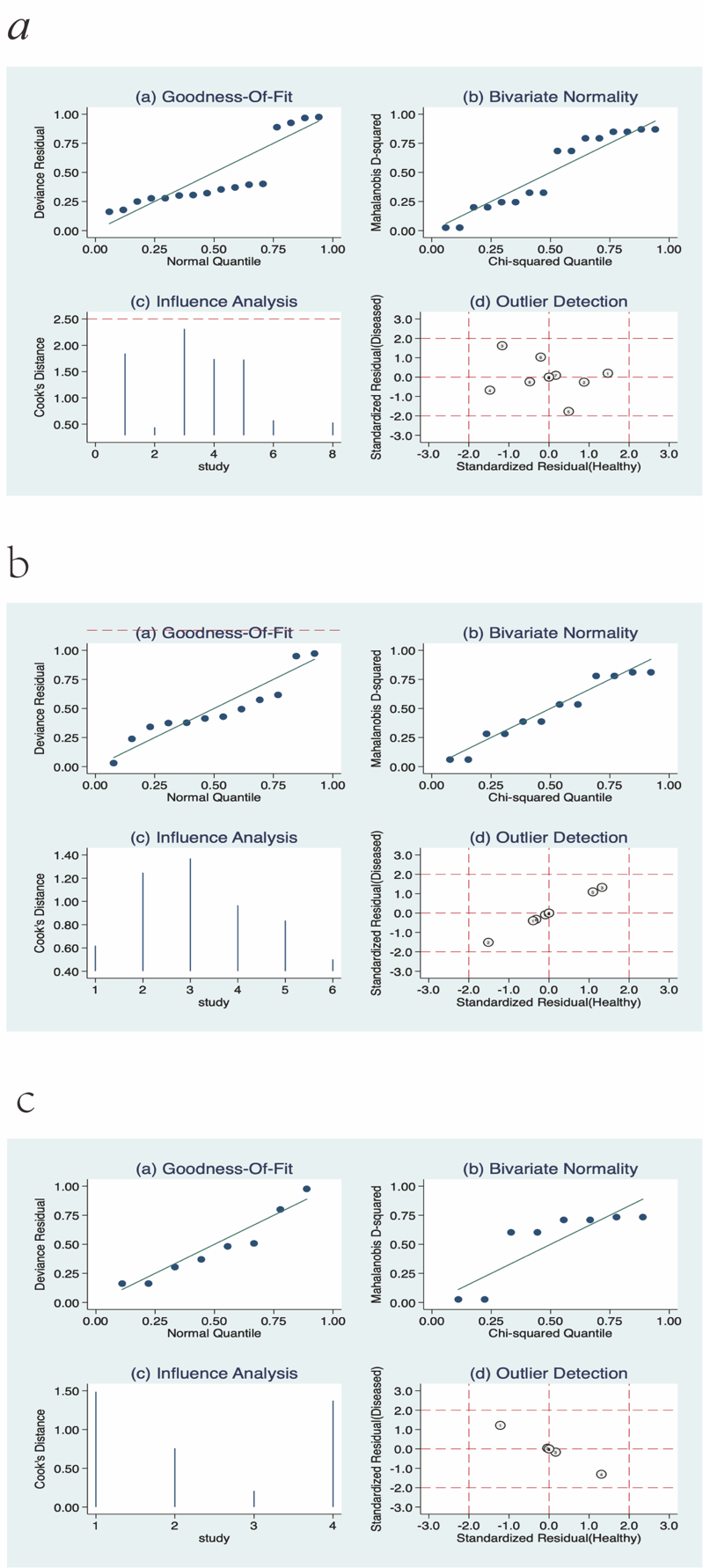


**Supplementary Figure 3.** Graphs for the assessment of the diagnostic value of lung ultrasound.

Abbreviations: LUQ, left upper quadrant; RUQ, right upper quadrant; LLQ, left lower quadrant; RLQ, right lower quadrant; LRP, likelihood ratio positive; LRN, likelihood ratio negative


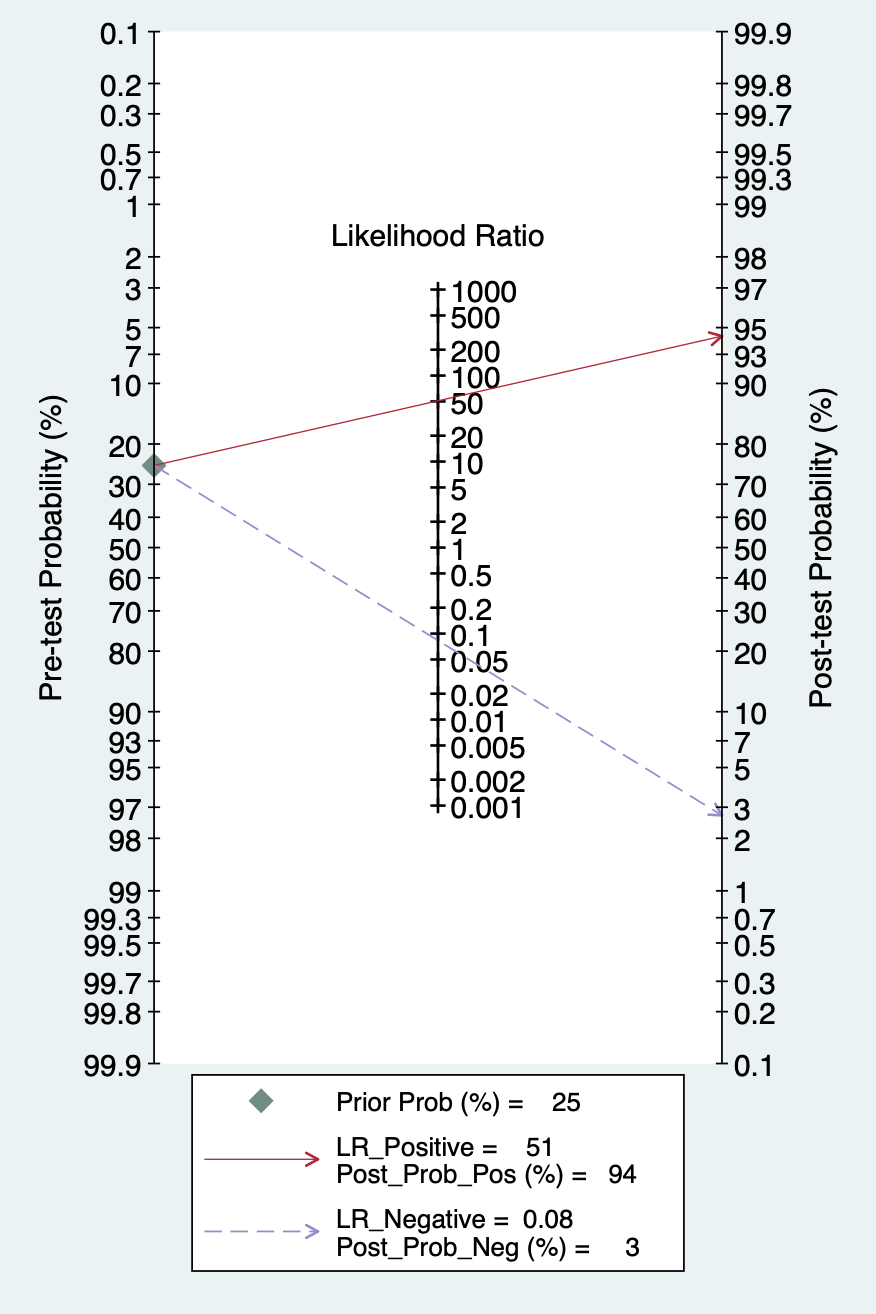

Supplement: Supplementary file 1 [file Data_Sheet_1.docx]
